# Four Cases of Penetrating Wounds of the Abdomen, with Visceral Lesions, and Wounds of Entrance above the Umbilicus

**Published:** 1900-05

**Authors:** Hugh M. Taylor

**Affiliations:** Richhond, Va. Professor of Practice of Surgery, University College of Medicine, Surgeon to Virginia Hospital, Etc.


### FOUR CASES OF PENETRATING WOUNDS OF THE ABDOMEN, WITH VISCERAL LESIONS, AND WOUNDS OF ENTRANCE ABOVE THE UMBILICUS.*

BY HUGH M. TAYLOR, M.D., RICHHOND, VA.

Professor of Practice of Surgery, University College of Medicine, Surgeon to Virginia Hospital, Etc.

The idea is often impressed that a bullet wound of the abdominal cavity in which the wound of entrance is above the umbilicus is very much less frequently attended by serious visceral lesions. I have had the opportunity of deciding the correctness of this conclusion in four cases of bullet wounds of the abdomen. In each instance the wound of entrance was above the umbilicus and in each I found multiple visceral lesions to treat.

This experience seems to warrant us in concluding that it is an error for us to have principles for our guidance in the treatment of penetrating wounds of the abdomen below the umbilicus which do not apply with equal force to similar wounds in which the wound of entrance is above the umbilicus. Notably in the last edition of the *American Text Book of Surgery* it is impressed that a bullet "which passes through the lower part of the abdomen from side to side, or obliquely, is almost sure to produce from four to fourteen perforations of the intestines; while the absence of dangerous visceral wounds may be inferred with some degree of probability if it crosses the abdominal cavity in antero-posterior direciion at or a little above the umbilical level." Such unqualified declarations, if allowed to pass unchallenged, cannot, we think, fail to have an influence opposed to early operative intervention in supra-umbilical penetrating wounds of the abdomen.

The following cases impress the fact that it is very much more important to try and infer the probable course of the bullet by ascertaining the position of the party shot to the one by whom the wound was inflicted:

---

*Read before the Tri-State Medical Association of the Carolinas and Virginia, during its session held in Charleston, S. C., February 20-22, 1900.

CASE I.—A young girl about seventeen years of age received a pistol wound through the left mammary gland. The wound of entrance was towards the axillary border of the gland and apparently passed through its base. I was informed that the girl at the time of the accident was seated in a low chair by the fire, and her father in taking a pistol from a tall mantel accidentally discharged it. A study of the probable course of the bullet would indicate a passage from above downward and from left to right across the abdominal cavity. When seen a few hours after the injury by her physician*, he found no evidence of shock or hemorrhage, or any manifestations of a serious injury, but recognizing the probable course of the bullet he brought her as quickly as possible to Richmond. When I saw her twelve hours later, there was no doubt of a serious intraperitoneal injury. The rapid pulse, elevation of temperature, muscular rigidity, distended abdomen and abolished peristalsis were conclusive evidence of peritoneal penetration with visceral lesion. A section disclosed a perforation in the diaphragm, two perforations in the transverse colon, five perforations of the small intestines and one in the cecum. The lesions were closed by mattress sutures; irrigation and wiping was practiced and multiple drainage was provided. The patient died in twenty-four hours from septic peritonitis. At the time of the operation twelve hours after the injury, the evidences of suppurative peritonitis were generally manifested. With our best efforts the operation was not performed in time to prevent septic peritonitis, and, as is usually the case, the operative intervention was important to cure it.

This case impresses that most important truth that the successful surgeon in such cases must be the one who gets into the abdomen and out of it just as quickly as is consistent with good work.

In this connection it may be well to recall that while an early operation and an operation of short duration is a great desideratum in all acute intraperitoneal infections, good work is equally essential, and we are admonished not to begin such work handicapped by poor assistants, etc. A post-mortem in this case satisfied us that no lesion had been overlooked and no leaking subsequent to the suturing had occurred.

*Dr Deitrick, of Hanover Co., Va.

CASE II.—This case equally impresses the probability of serious multiple intraperitoneal lesion with supra-umbilical wound of entrance, and the further fact that the probable course of the bullet is an essential consideration.

The case was a homicide, and as the prisoner was acquitted, we presume the defense proved, as was claimed, that the party shot had the prisoner down beating him. However that may be, we found a wound through the left rectus muscle an inch or more above the umbilicus. A section disclosed nine perforations of the intestines and nine of the mesentary. This patient was not brought to Richmond until twenty-four hours after being wounded, and in that time gross manifestations of intraperitoneal infections were evident. This case is further interesting in that the patient lived for eight days after the operation and died from acute infection and sloughing of the abdominal incision. While septic peritonitis existed at the time of the operation, thorough irrigation, wiping and multiple drainage seemed to have arrested infection from that focus, and I think the patient would have recovered but for the sloughing of the abdominal incision throughout its length and depth. The abdominal incision was presumably infected either by the septic contents of the peritoneal cavity. at the time of the operation or subsequently through contact with the strips of gauze drainage. Localized peritonitis around the abdominal wound occurred, coils of intestines protruded into the open abdominal wound and became adherent and shut off the focus of infection from the general peritoneal cavity. In this case I tried, without appreciable benefit, antistreptococcus serum (Parke, Davis & Co.). I think this patient's life was prolonged by the free use of saline solution under the skin, and by the exhibition of two drops of croton oil between layers of glycerine to relieve distension in the adherent intestines, and certainly the patient's comfort was greatly promoted by irrigation of the stomach to relieve the gulping vomiting of general sepsis.

A post-mortem revealed that there had been no leaking into the peritoneal cavity, the sutured points were all closed and the general peritoneal sac was practically dry. The bullet on entering above the umbilicus had passed from above downward, through the whole length of the peritoneal cavity to the pelvis, and was found loose in Douglas's cul-de-sac, having probably rebounded after striking somewhere on the bony pelvic wall.

CASE III.—A third case of supra-umbilical wound was received while the injured man was standing on the ground, and the party who did the shooting was on top of a passing box- or coal-car. The wound of entrance was above and to the right of the umbilicus.

A section disclosed a perforation of the greater curvature of the stomach and also a perforation of the free margin of the liver. The position of the parties was sufficient to infer that the bullet had passed through the peritoneal cavity from right to left and from above downward, as it lodged in the left lumbar muscles.

A section six hours after the reception of the injury was made, the stomach lesion was sutured, the wound in the liver plugged with a long strip of gauze, one end of which was brought through the abdominal incision, and the man made an uneventful recovery.

CASE IV.—A policeman was shot while in pursuit of a prisoner. The wound of entrance was considerably above and to the left of the umbilicus. The bullet passed through the right lobe of the liver, the right meso-colon and lodged in the muscles of the right lumbar region. In spite of the fact that prompt operative intervention was resorted to this patient bled to syncope, and I attributed his death, which occurred in twelve hours, to acute anemia incident to hemorrhage. Even when the wound of entrance is below the umbilicus we have no symptom or combination of symptoms which enable us to differentiate between the penetrating and non-penetrating wound *with* from one *without* visceral lesion. The occurrence of a number of symptoms such as muscular rigidity, abolished peristalsis and shock with reaction and succeeding shock are strongly suggestive, but not confirmatory, and are of especial value, as when they do occur they are early manifestations. There seems to be a consensus of opinion that there are no symptoms of intra-abdominal lesion sufficiently uniform in their expression to enable us to make an early diagnosis of penetration with visceral lesion. Decidedly more exact information may be obtained by a study of the probable course of the bullet, by a careful dissection of the tubular wound tract, and on finding it a penetrating wound, by an exploratory celiotomy. By these means only can we ascertain beyond all question the extent of the morbid lesion in time to do preventive and conservative surgery. The late symptoms, *i. e.*, those incident to septic peritonitis, are of course gross in their manifestation, and any one can make the diagnosis; but with their occurrence we have lost the key to success, an early operation to prevent acute intraperitoneal infection.

I am conscious of the fact that able clinicians impress the idea that we not infrequently have penetration without visceral lesion. I admit such a possibility; I doubt its frequency.* Operative intervention upon all sorts of cases has saved sixty-seven per cent., and an early operation in a series of cases should give even better results. This showing is, we think, a sufficient proof that masterly inactivity until the occurrence of symptoms of acute intraperitoneal infection is bad surgery.

*"In 21 of 253 operations neither perforation nor serious hemorrhage was found. It is to be doubted whether so large a number of traced penetrating bullets could fail of more serious injury, and there is a presumption of faulty diagnosis in some of them. However, they all recovered; so the operation did no harm."—*Grant.*

